# Supplementary material for: The seventh pandemic of cholera in Europe revisited by microbial genomics
Source: Nat Commun. 2020 Oct 22;11:5347. doi: 10.1038/s41467-020-19185-y (PMC7582136; doi:10.1038/s41467-020-19185-y)
Supplement: Supplementary file 1 — Supplementary Information [file 41467_2020_19185_MOESM1_ESM.pdf]

Supplementary Information

The seventh pandemic of cholera in Europe revisited by microbial genomics

Mihaela Oprea, Elisabeth Njamkepo, Daniela Cristea, Anna Zhukova, Clifford G. Clark, Anatoly N. Kravetz, Elena Monakhova, Adriana S. Ciontea, Radu Cojocaru, Jean Rauzier, Maria Damian, Olivier Gascuel, Marie-Laure Quilici, François-Xavier Weill

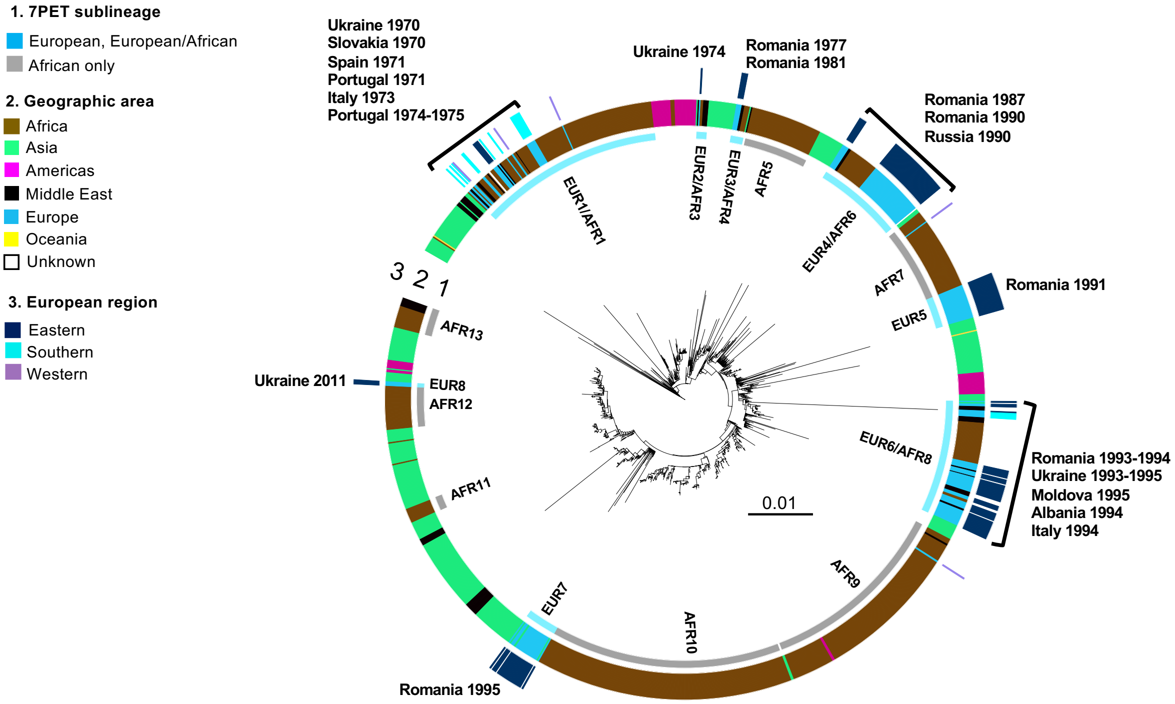

Supplementary Fig. 1

Population structure of the 1,324 seventh pandemic *V. cholerae* O1 El Tor isolates analyzed in this study

Maximum likelihood phylogeny inferred from 13,544 single-nucleotide variants (SNVs). A6 was used as an outgroup. The colored rings represent: (1) the relevant seventh pandemic *V. cholerae* O1 El Tor (7PET) sublineages, the names of which are indicated perpendicularly to the inner ring, (2) the geographic origin of the isolates, and (3) the countries of origin for the European isolates. The scale bar indicates the number of substitutions per variable site (SNVs).

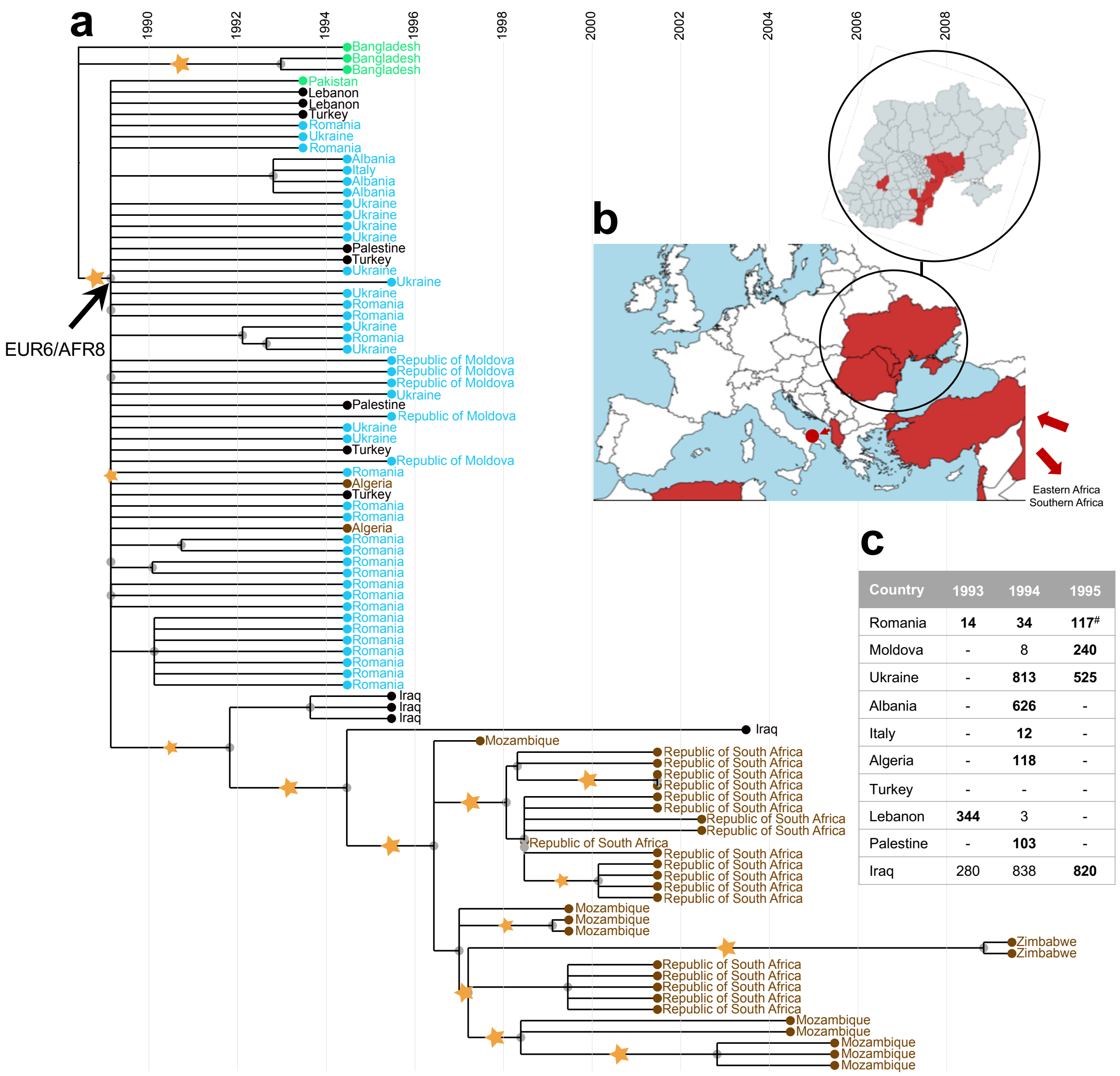

Country	1993	1994	1995
Romania	**14**	**34**	**117**#
Moldova	-	8	**240**
Ukraine	-	**813**	**525**
Albania	-	**626**	-
Italy	-	**12**	-
Algeria	-	**118**	-
Turkey	-	-	-
Lebanon	**344**	3	-
Palestine	-	**103**	-
Iraq	280	838	**820**

Supplementary Fig. 2

Geographic expansion of the seventh pandemic *V. cholerae* O1 EUR6/AFR8 sublineage

a, Timed phylogeny showing the seventh pandemic *V. cholerae* O1 El Tor (7PET) isolates of the EUR6/AFR8 sublineage. The colors at the end indicate the geographic location from which the isolates were obtained (same colors as in Supplementary Fig. 1 (ring 2)). Nodes supported by bootstrap values ≥ 0.9 are indicated by orange stars. **b,** Countries of the Black Sea region and the Mediterranean Basin, in which the EUR6/AFR8 sublineage was isolated, are shown in red. A more precise view of the affected regions of Romania, Ukraine and Moldova is provided in the magnification. The maps were created with mapchart.net (https://mapchart.net/feedback.html). **c,** Number of cholera cases reported to the WHO between 1993 and 1995. The numbers of cases are indicated in bold typeface if the isolates from the year and country concerned were included in this study. # In 1995, the Romanian isolates belonged to another 7PET sublineage, EUR5.

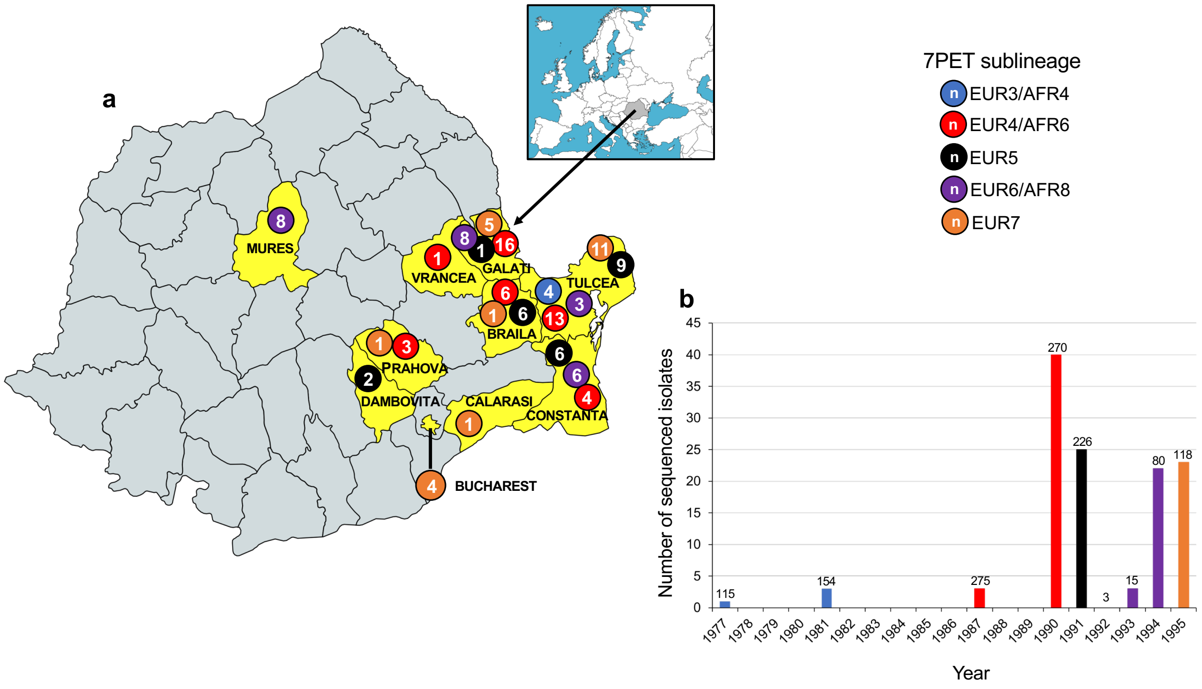

Supplementary Fig. 3

Seventh pandemic *V. cholerae* O1 sublineages observed in Romania between 1977 and 1995

a, Geographic location (at region level) and phylogenetic grouping of the 120 Romanian seventh pandemic *V. cholerae* O1 El Tor (7PET) isolates studied here. The inset indicates, in gray, the position of Romania within Europe. The names of the affected regions are indicated. "n" indicates the number of isolates per sublineage. The maps were created with mapchart.net (https://mapchart.net/feedback.html). **b,** The graph shows the results of phylogenetic grouping by year of isolation. The number of cholera cases reported in Tulcea (1977, 1981, 1987) or throughout the entire country (1990-1995) are indicated above the graph. In 1993, one of the 15 cases was notified as an imported case, whereas, in 1994, 46 of the 80 cases were identified as imported cases.

Supplementary Table 1: GenBank accession numbers used for our analyses

Target	Strain	Accession no.	Accessible at:	Coordinates
rfb O1	*Vibrio cholerae* O1 N16961	AE003852	https://www.ncbi.nlm.nih.gov/nuccore/AE003852.1	258103-258294
rfb O139	*Vibrio cholerae* O139 AI-1837	Y07786	https://www.ncbi.nlm.nih.gov/nuccore/Y07786	12288-12736
ctxB	*Vibrio cholerae* O1 N16961	AE003852	https://www.ncbi.nlm.nih.gov/nuccore/AE003852.1	1566967-1567341
wbeT	*Vibrio cholerae* O1 VX44945	JF284685	https://www.ncbi.nlm.nih.gov/nuccore/JF284685	1-953
VSP-II	*Vibrio cholerae* O1 N16961	AE003852	https://www.ncbi.nlm.nih.gov/nuccore/AE003852.1	523156-550021
WASA-1	*Vibrio cholerae* IEC224	CP003330	https://www.ncbi.nlm.nih.gov/nuccore/CP003330.1	1608668-1648059
GI-15	*Vibrio cholerae* O1 V060002	AP018677	https://www.ncbi.nlm.nih.gov/nuccore/AP018677.1	844-29014
Tn*7*	*Shigella sonnei* Ss046	CP000038	https://www.ncbi.nlm.nih.gov/nuccore/CP000038	4098721-4113720
ICE*Vch*Ind5	*Vibrio cholerae* O1 Ind5	GQ463142	https://www.ncbi.nlm.nih.gov/nuccore/GQ463142.1	1-97952
ICE*Vch*Ban9	*Vibrio cholerae* O1 MJ-1236	CP001485	https://www.ncbi.nlm.nih.gov/nuccore/CP001485.1	2959802-3065926
ICE*Vch*Ind4	*Vibrio cholerae* O139 Ind4	GQ463141	https://www.ncbi.nlm.nih.gov/nuccore/GQ463141.1	1-95326
gyrA	*Vibrio cholerae* O1 N16961	AE003852	https://www.ncbi.nlm.nih.gov/nuccore/AE003852.1	1330207-1332891
parC	*Vibrio cholerae* O1 N16961	AE003852	https://www.ncbi.nlm.nih.gov/nuccore/AE003852.1	2603309-2605594
VC_0715	*Vibrio cholerae* O1 N16961	NC_002505	https://www.ncbi.nlm.nih.gov/nuccore/NC_002505.1	768551-769273
VC_A0637	*Vibrio cholerae* O1 N16961	NC_002506	https://www.ncbi.nlm.nih.gov/nuccore/NC_002506.1	571824-572477
vprA (VC1320)	*Vibrio cholerae* O1 N16961	NC_002505	https://www.ncbi.nlm.nih.gov/nuccore/NC_002505.1	1404377-1403673

The sequences for ICE*Vch*Ban11 and ICE*Vch*Ind6 were kindly provided by Matteo Spagnoletti and have been deposited in FigShare [https://doi.org/10.6084/m9.figshare.5422912]

Supplementary Notes

French autochthonous sporadic cases

Three sporadic autochthonous cholera cases documented in France in 1970, 1971, and 1974 were caused by seventh pandemic *V. cholerae* O1 El Tor (7PET) serotype Ogawa isolates from the EUR1/AFR1 sublineage. The first case[1] occurred in a 65-year-old woman living 40 km south-west of Paris, in October 1970. This patient had never traveled and had no contact with anyone from an area in which cholera was endemic. No secondary cases occurred. Her isolate (CNRVC930079) clustered within the lower part of EUR1/AFR1, with isolates from West Africa collected in 1970. It will never be possible to determine the origin of contamination in this particular case, but an unknown link with a patient or carrier from West Africa would appear likely, given the colonial history of France. Rondle *et al.*[2] also suggested the possibility of infection via aircraft effluent.

The second case occurred in September 1971, in a 29-year-old woman living in Hendaye, close to the Spanish border (unpublished). This patient made frequent trips to Spain. Her isolate (CNRVC950546) clustered within the ancestral part of the EUR1/AFR1 sublineage, like the Spanish and Portuguese isolates collected in 1971.

The third case[3] occurred in a 19-year-old Algerian living with 10 relatives in an urban slum in Marseilles, in September 1974. In addition to this severe case, one of the patient's relatives suffered from mild cholera and nine others were carriers of the bacterium. Hygiene measures were implemented and chemoprophylaxis by sulfonamides was administered to the other 180 inhabitants of the slum. No secondary cases occurred. The isolate obtained from the most severely affected case (CNRVC950418) was

sequenced and clustered with four Algerian isolates collected in 1975. These five isolates had a specific synonymous SNV (G → T) at nucleotide position 572532 in common. Contamination probably occurred through the transitory passage through this slum of numerous migrants from the other side of the Mediterranean Sea.

The genomic analysis also showed that the isolates from two non-imported cases occurring in France in 1993 and 1996 belonged to sublineages AFR7 and AFR9, which were circulating in Africa at the time. These two cases were African immigrants living in France who had not traveled before disease onset. For the second case, the consumption of fresh sorrel imported from West Africa was suggested as a possible cause of the disease[4]. The genomic clustering was consistent with the region of origin of these African immigrants: North Africa for the first case and West Africa for the second.

7PET outbreak isolates not studied

In 1979, Spain reported 267 cases[5], but no isolates from this year were available for this study. In the same year, Algeria reported its highest number of cholera cases (n=2513) since 1971. Three of our isolates from Algeria for 1982-1983 and one from Tunisia for 1982 belonged to the distal part of the EUR1/AFR1 sublineage. It is, therefore, tempting to speculate that the 7PET isolates obtained in Spain in 1979 probably belonged to the EUR1/AFR1 sublineage.

A cholera outbreak occurred in the southern part of Ukraine in 1991, with 75 cases and no deaths reported to the WHO[5], whereas a published study on this outbreak mentioned approximately 300 cases and carriers[6]. A single isolate from this outbreak (95-0743) has been described[6,7]. We sequenced this isolate and found that it belonged to

EUR6/AFR8, together with Ukrainian isolates from 1994 and 1995. It was resistant to cotrimoxazole, due to the presence of both GI-15 and IC*Vch*Moz10/IC*Vch*Ban9. However, four representative isolates of this 1991 cholera outbreak stored in the collection of the Rostov-on-Don Research Anti-Plague Institute, in the Russian Federation, did not display resistance to cotrimoxazole (E. Monakhova, personal communication). We searched for an original culture of 95-0743 or another independent representative isolate (those mentioned above could not be added to our study due to current *V. cholerae* regulations in the Russian Federation), to rule out a possible mix-up with the 1994-1995 isolates in previous studies[6,7]. This search was unsuccessful and we therefore removed 95-0743 from our analysis, due to the impossibility of resolving doubts about this unique isolate. Only the sequencing of several new independent isolates would make it possible to identify reliably the 7PET sublineage responsible for the 1991 Ukrainian cholera outbreak. The two most likely possibilities are EUR4/AFR6 and EUR5, which were circulating in neighboring Romania in 1990 and 1991, respectively.

Between 1965 and 1989, 10,723 cases of cholera and carriers in total were reported by 11 Soviet Republics, including 7,720 for Russia[8]. As only two isolates from the Russian Federation were available for this study, a large-scale whole-genome sequencing analysis of 7PET isolates collected there since 1965 would be of interest to shed light on the regional circulation of the causal agent of cholera across this vast territory and to define more precisely the routes of propagation from South Asia to Eastern Europe.

Supplementary References

1. Deparis, M., Gallut, J., Scavizzi, M., Rykner. & G, Auzepy, P. Remarques cliniques et épidémiologiques à propos d'un cas sporadique (vibrion El Tor souche Ogawa) observé à Paris. *Bull. Acad. Natl Med.* **155,**135-141 (1971).
2. Rondle, C.J., Ramesh, B., Krahn, J.B. & Sherriff R. Cholera: possible infection from aircraft effluent. *J. Hyg. (Lond)* **81,** 361-371 (1978).
3. Gallais, H., Cabrajas A., Gevaudan, P. & Casanova, P. Epidémiologie d'une émergence du choléra à Marseille. *Med. Mal. Infect*. **4,** 223-226 (1975).
4. Infuso, A. et al. Choléra autochtone à Paris : une observation. *Presse Med*. **27,** 202-2044 (1998).
5. Cholera, Global Health Observatory data repository, accessible at: https://apps.who.int/gho/data/node.main.174?lang=en (last accessed September 24th 2020)
6. Clark, C.G., Kravetz, A.N., Alekseenko, V.V., Krendelev, Yu.D. & Johnson, W.M. Microbiological and epidemiological investigation of cholera epidemic in Ukraine during 1994 and 1995. *Epidemiol. Infect*. **121,** 1-13 (1998).
7. Clark, C.G. et al. Investigation of the 1994-5 Ukrainian *Vibrio cholerae* epidemic using molecular methods. *Epidemiol. Infect*. **121,** 15-29 (1998).
8. Narkevich, M.I. et al. The seventh pandemic of cholera in the USSR, 1961-89. *Bull. World Health Organ*. **71,** 189-196 (1993).
