# Supplementary material for: The seventh pandemic of cholera in Europe revisited by microbial genomics
Source: Nat Commun. 2020 Oct 22;11:5347. doi: 10.1038/s41467-020-19185-y (PMC7582136; doi:10.1038/s41467-020-19185-y)
Supplement: Supplementary file 3 — Descriptions of Additional Supplementary Files [file 41467_2020_19185_MOESM3_ESM.pdf]

Descriptions of Additional Supplementary Files

Supplementary Data 1

Description: Vibrio cholerae O1 isolates and genomes used in this study. The following are shown: name, year and country of isolation, source, epidemiological information, biotype, serotype, ctxB and VSP-II alleles, genomic wave, sublineage, antibiotic resistance phenotype, antibiotic resistance gene and element, other typing results, identification as an outlier according to LSD2, GenBank and EBI-ENA accession numbers.
