# Supplementary material for: The seventh pandemic of cholera in Europe revisited by microbial genomics
Source: Nat Commun. 2020 Oct 22;11:5347. doi: 10.1038/s41467-020-19185-y (PMC7582136; doi:10.1038/s41467-020-19185-y)
Supplement: Supplementary file 5 — Reporting Summary [file 41467_2020_19185_MOESM5_ESM.pdf]

nature research

The seventh pandemic of cholera in Europe revisited by microbial genomics (NCOMMS-20-24076-A)

Corresponding author(s): Weill

Last updated by author(s): Sep 24, 2020

Reporting Summary

Nature Research wishes to improve the reproducibility of the work that we publish. This form provides structure for consistency and transparency in reporting. For further information on Nature Research policies, see our Editorial Policies and the Editorial Policy Checklist.

Statistics

For all statistical analyses, confirm that the following items are present in the figure legend, table legend, main text, or Methods section.

n/a	Confirmed	
x	☐	The exact sample size (*n*) for each experimental group/condition, given as a discrete number and unit of measurement
x	☐	A statement on whether measurements were taken from distinct samples or whether the same sample was measured repeatedly
x	☐	The statistical test(s) used AND whether they are one- or two-sided<br>*Only common tests should be described solely by name; describe more complex techniques in the Methods section.*
x	☐	A description of all covariates tested
x	☐	A description of any assumptions or corrections, such as tests of normality and adjustment for multiple comparisons
x	☐	A full description of the statistical parameters including central tendency (e.g. means) or other basic estimates (e.g. regression coefficient) AND variation (e.g. standard deviation) or associated estimates of uncertainty (e.g. confidence intervals)
x	☐	For null hypothesis testing, the test statistic (e.g. *F*, *t*, *r*) with confidence intervals, effect sizes, degrees of freedom and *P* value noted<br>*Give P values as exact values whenever suitable.*
x	☐	For Bayesian analysis, information on the choice of priors and Markov chain Monte Carlo settings
x	☐	For hierarchical and complex designs, identification of the appropriate level for tests and full reporting of outcomes
x	☐	Estimates of effect sizes (e.g. Cohen's *d*, Pearson's *r*), indicating how they were calculated

Our web collection on statistics for biologists contains articles on many of the points above.

Software and code

Policy information about availability of computer code

Data collection	Excel version 15.41
Data analysis	Snippy v4.1.0/BWA-MEM v0.7.13 (https://github.com/tseemann/snippy), Snippy v4.1.0/Freebayes v1.1.0 (https://github.com/tseemann/snippy), SPAdes version 3.1.0 (http://cab.spbu.ru/files/release3.1.0/manual.html), BLAST version 2.2.26 (https://bioweb.pasteur.fr/packages/pack@blast@2.2.26), ResFinder version 3.1.0 (https://cge.cbs.dtu.dk/services/ResFinder/), PlasmidFinder version 2.0.1 (https://cge.cbs.dtu.dk/services/PlasmidFinder/), Gubbins version 2.3.4 (https://bioweb.pasteur.fr/packages/pack@gubbins@2.3.4), RAxML version 8.0.20 (https://cme.h-its.org/exelixis/web/software/raxml/), iTOL version 5 (https://itol.embl.de), LSD2 version 1.6.5 (https://github.com/tothuhien/lsd2), PastML version 1.9.29.2 (https://github.com/evolbioinfo/pastml), fasta_to_fastq.pl (https://github.com/ekg/fasta-to-fastq/blob/master/fasta_to_fastq.pl)

For manuscripts utilizing custom algorithms or software that are central to the research but not yet described in published literature, software must be made available to editors and reviewers. We strongly encourage code deposition in a community repository (e.g. GitHub). See the Nature Research guidelines for submitting code & software for further information.

Data

Policy information about availability of data

All manuscripts must include a data availability statement. This statement should provide the following information, where applicable:
- Accession codes, unique identifiers, or web links for publicly available datasets
- A list of figures that have associated raw data
- A description of any restrictions on data availability

Short-read sequence data were submitted to the European Nucleotide Archive (ENA), under study accession number PRJEB38484 [https://www.ebi.ac.uk/ena/

browser/view/PRJEB38484], and the genome accession numbers are provided in Supplementary Data 1. The whole-genome alignment for the 1,324 genomes and other files that support the findings of this study have been deposited in FigShare [https://doi.org/10.6084/m9.figshare.5422912]. The phylogeny and associated metadata have been uploaded to Microreact for interactive viewing [https://microreact.org/project/choleraeurope]. The GenBank accession numbers used for our analyses are shown in Supplementary Table 1.

Field-specific reporting

Please select the one below that is the best fit for your research. If you are not sure, read the appropriate sections before making your selection.

- [x] Life sciences
- [ ] Behavioural & social sciences
- [ ] Ecological, evolutionary & environmental sciences

For a reference copy of the document with all sections, see nature.com/documents/nr-reporting-summary-flat.pdf

Life sciences study design

All studies must disclose on these points even when the disclosure is negative.

Sample size	No statistical analysis was carried out. We have sequenced all viable V. cholerae O1 strains recovered from European autochthonous cases reported by the World Health Organization (ref.4 accessible at https://apps.who.int/gho/data/node.main.174?lang=en) and available in the collections of the French National Reference Center for Vibrios and Cholera, Institut Pasteur, Paris, France and the Cantacuzino Institute, Bucharest, Romania. We have also sequenced 14 isolates out of 35 isolates collected during the 1991 and 1994-1995 Ukrainian cholera outbreaks and stored at the Public Health Agency of Canada. These 14 sequenced isolates were selected to cover the broadest diversity in terms of sources, geographical locations, data and disease outcomes (according to Clark et al. Infect Epidemiol 1998). We have also included 1,168 seventh pandemic V. cholerae El Tor (7PET) genomic sequences from previously published studies (Weill et al. Science 2017 and Weill et al. Nature 2019) in order to place the European isolates into the global phylogenetic context of 7PET.
Data exclusions	Six European V. cholerae genomes were excluded because they did not belong to the 7PET population. One Ukrainian genome (95-0743) was excluded due to the impossibility of resolving doubts about this unique isolate representing the 1991 Ukrainian cholera outbreak. This was extensively discussed in the Supplementary Information.
Replication	The maximum likelihood phylogenetic tree was successfully replicated four times.
Randomization	We used randomization for the geographic predictions of the most recent common ancestors (MRCAs) of European clades. We performed such reconstruction under the same settings for five trees obtained by pruning some of the leaves corresponding to overrepresented European regions from the full time-tree. For each pruned subtree, we retained two (randomly selected) of 36, two of 20, and two of 40 leaves from those sampled in Romania in 1990, 1991, and 1993-1995, respectively, four of 18 leaves from those sampled in Ukraine or Moldova in 1993-1995, all the leaves sampled in India in 1990 (n=3) and 1991 (n=3), and all the leaves sampled from India and Pakistan in 1993-1995 (n=9). This procedure made it possible to bring the sampling proportions for these countries closer to the proportions of reported cases and these sampling variations did not change the geographic prediction for the MRCAs of EUR4/AFR6, EUR5, EUR6/AFR8.
Blinding	Not applicable.

Reporting for specific materials, systems and methods

We require information from authors about some types of materials, experimental systems and methods used in many studies. Here, indicate whether each material, system or method listed is relevant to your study. If you are not sure if a list item applies to your research, read the appropriate section before selecting a response.

Materials & experimental systems

n/a	Involved in the study	
[x]	[ ]	Antibodies
[x]	[ ]	Eukaryotic cell lines
[x]	[ ]	Palaeontology and archaeology
[x]	[ ]	Animals and other organisms
[x]	[ ]	Human research participants
[x]	[ ]	Clinical data
[x]	[ ]	Dual use research of concern

Methods

n/a	Involved in the study	
[x]	[ ]	ChIP-seq
[x]	[ ]	Flow cytometry
[x]	[ ]	MRI-based neuroimaging
